# What do you think of an unusual axillary mass?

**DOI:** 10.11604/pamj.2017.28.309.3092

**Published:** 2017-12-13

**Authors:** Hafsa Benzzi, Majda Askour, Afaf Elouazani, Mouna Bouaddi, Badreddine Hassam, Amar Saidi

**Affiliations:** 1 Dermatology Department, Mohamed V University, Rabat, Morocco; 2 Pathology Center United Nations, Rabat, Morocco

**Keywords:** Axillary mass, skin, apocrine adenocarcinoma

## Abstract

Skin apocrine carcinoma is a rare malgnancy of epidermal adnexa, most frequent in axillary seat, where apocrine sweat gland are abundant, the neoplasm can arise in groin, anogenital, lips, eyelid, characterized by a plate or surface area of nodules hummocky. Etiology and incidence are not known. The prognosis is influenced by the risk of locoregional recurrence and metastatic evolution. We describe the case of 61-year-old man who presented a left axillary slow-growing mass since 2 years ago. The cutaneous biopsy objectified an apocrine adenocarcinoma. The paraclinic exams performed to detect primary breast were tumor negative, first step before confirming the diagnosis. Standard treatment is surgical excision with margins of 2 to 3cm for local tumor, for apocrine adenocarcinoma regional lymph node dissection if nodes were clinically positive is wide surgical excision. This kind of tumour is chemoresistant. In this case, adjuvant chemotherapy was indicated, before surgery to reduce tumoral volume. This case illustrates the importance clinicopathological correlation of skin cancer, particularly apocrine one. Clinical particularity and careful analyses histology helps diagnosis approach.

## Introduction

Skin apocrine carcinoma is a rare malignancy of epidermal adnexa, most frequent in axillary seat, where apocrine sweat gland are abundant, the neoplasm can arise in groin, anogenital, lips, eyelid, characterized by a plate or surface area of nodules hummocky. Etiology and incidence are not known. The prognosis is influenced by the risk of locoregional recurrence and metastatic evolution [1]. The slow evolution, painless charatacter leads to discover the tumor at systemic dissemination with locally invasive stage. The differential diagnosis between CAC and axillary skin metastasis adenocarcinoma, particularly breast is sometimes difficult. In the following report we present the case of a 61-year-old man with apocrine adenocarcinoma of the left axillary area with local lymph and distant metastases, which illustrates the difficulty.

## Patient and observation

**Figure 1:**
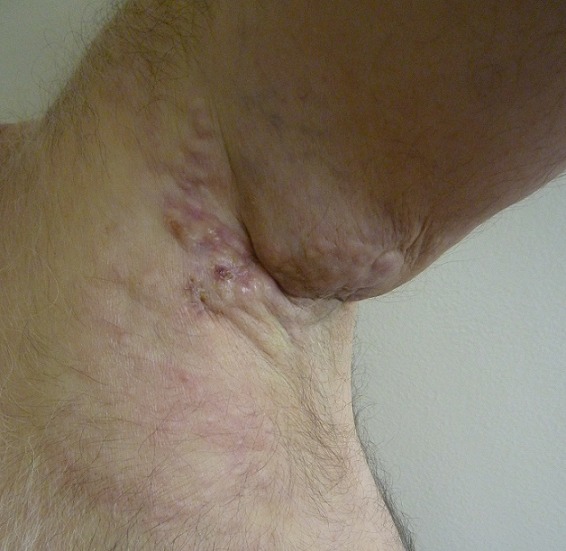
An axillary hummoky tumor

**Figure 2:**
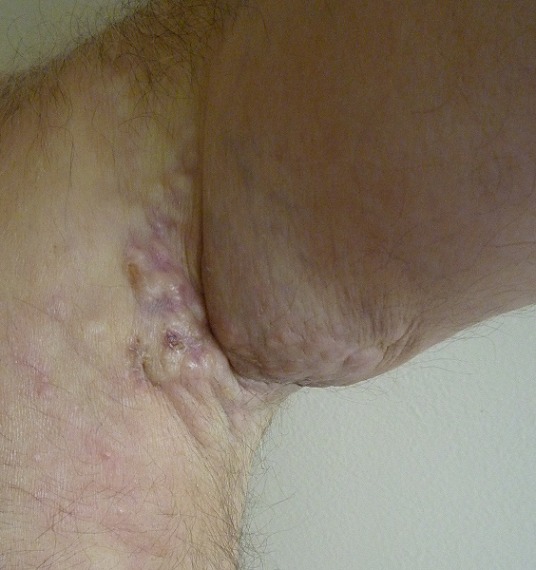
Burgeoning aspect of the tumor

**Figure 3:**
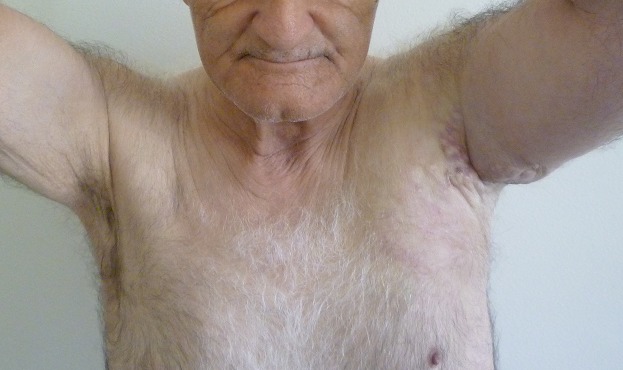
Edema of the upper limb

We describe the case of 61-year-old man without a medical history who consulted dermatology department presenting a left axillary slow-growing mass (Figure 1, Figure 2) since 2 years ago, painless at first, becoming painful since 6 months that conducts the patient to consult. Physical examination objectified a hummocky plate full of nodules measuring 10-6cm, erythematous, purple color, painful at mobilization, adherent, the plate is infiltrating surrounding tissue, there was no bleeding or serious discharge. The member was oedematous (Figure 3), superficial venous maze, without neither palpable mass of breast nor supernumerary nipple. There was homolateral nodes individualized clinically and the somatic examination was normal. The cutaneous biopsy objectified an apocrine adenocarcinoma. The paraclinic exams performed to detect primary breast were tumor negative. A thorough systemic workup for metastatic disease have performed, tomography showed lungs and nodes, scintigraphy showed bones metastases. In conlcusion, the patient presented an axillary adenocarcinoma apocrine with node, bone and lung metastases. After multi-disciplinary concertation a polychimiotherapy was indicated.

## Discussion

CAC constitutes a rare cutaneous adenocarcinoma, commonly seen in the axilla [**1**]. The primary cutaneous apocrine carcinomas are malignant adnexal tumor that develops in skin areas rich in apocrine glands. Presumptive progenitor cells for apocrine differentiation may be present along the lines joining the axillae, areolae and anogenital and they may be responsible for giving rise to some examples of extramammary Paget´s disease [2]. They arise in the form of plaques or nodules hummocky more or less confluent, painless. Evolution is the more often indolent, slowly progressive, but can be aggressive, with a risk of local recurrence or metastatic patterns including the pulmonary, brain and bone, which can lead to death [3]. One hundred cases of ACC [4,5], were described in literature, mainly located in the axillary level, but other locations are possible especially on the scalp, forehead, eyelids the upper lip, cheeks, the pubis, the nipple and fingers [6].

Before treating, the difficulty of differential diagnosis between histological CAC and axillary metastasis of lobular carcinoma breast was recently highlighted [7] or supernumerary breast carcinoma, some malignant tumor of sweat glands, the whole interest of a thorough clinical and histological study. Axilla is undoubtedly the most frequent location of apocrine tumor due to high density of apocrine glands, other locations are possible especially on the scalp, forehead, eyelids, the upper lip, cheeks, the pubis, the nipple and fingers [8]. Clinically, the macroscopic aspect is not specific, nodules or plaques, initially flesh-colored, with a slow growth.

**Figure 4:**
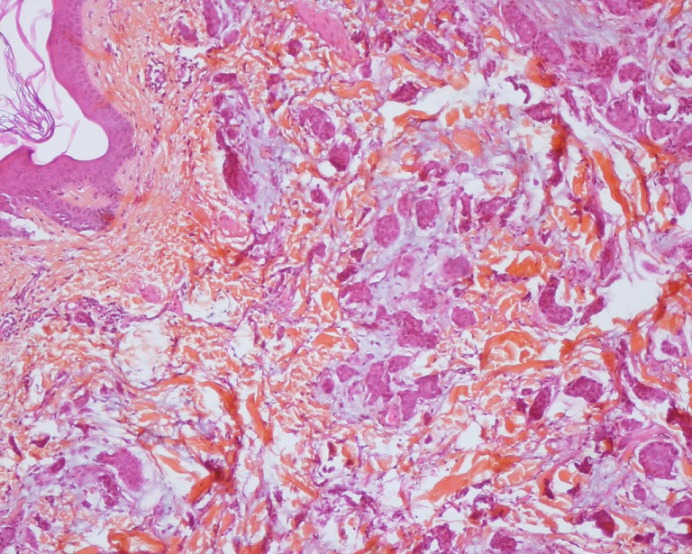
Carcinomatous proliferation separating the collagen fibers of the deep dermis: it is arranged in tubes, in wires and of variable size in lobules (x4)

**Figure 5:**
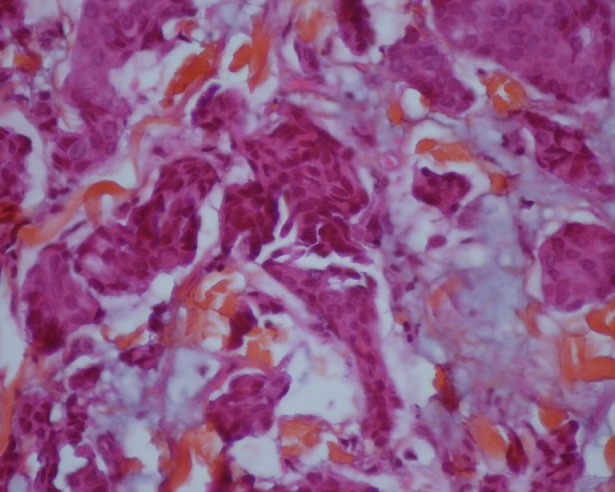
Cells with increased volume in places, often vesicular nucleus and nucleolus and a hyperchromic also eosinophilic granular cytoplasm (x10)

To discuss diagnostic of cutaneous metastases adenocarcinoma and therefore, we need imperatively complete morphological examination. Histologicaly the ACC are organized in tubular and papillary structures or massive basophils (Figure 4, Figure 5), the diagnosis of apocrine tumor has been mentied in presence of decapitation secretion images in a cystic structure or projections papillary [9], like our patient who had a sheet of cell floating in mucus, the nucleus is large, irregular and abundant cytoplasm. Sometimes there is variation in patterns of tumors. Obaidat et al. [10] described it as a “composition of tubular and ductal structures, papillary projections, subcutis infiltration, with necrotic area and decapitation of secretion”. In the other hand, Zelger et al. [1] reported that the histology of CAC is a “cobblestone pattern” of epithelium, vacuoles along the apical end of the epithelium, with variety of cell types and apocrine secretion. So both of descriptions insist on decapitation of secretion in eosinophilic epithelial cells and the second argument is attachment of neighbouring follicular epithelium.

The presence of a positive immunostaining may help in diagnosis but is not a sufficient argument. The GCDFP-15 is expressed specifically by apocrine epithelium but may still be any positive mammary adenocarcinoma in differentiation associated with apocrine gland secretion and carcinomas of the salivary glands [11]. In fact, many of the reported CACs have shown weak and focal expression of GCDFP-15 or have failed to demonstrate any expression of the marker [7]. A immunostaining with anti-herceptin-2 [Her-2] can be used because it is sometimes positive in skin metastases a mammary adenocarcinoma, whereas carcinomas primary cutaneous apocrine or eccrine do not set the anti-Her-2 [12]. Hormone receptors, however, are not discriminatory to distinguish CAC and metastasis lobular breast carcinoma.

In the past, negativity of ER and PR with positive expression of androgen receptor (AR) has shown to be relevant criteria for diagnosis of CAC [13]. Klein et al. [14] also suggested that the lack of expression of ER and PR is more important from a practical point of view than positivity of AR. In contrast, Robson et al. [15] demonstrated that 62% were positive for ER, 60% were positive for PR and 36% were negative for AR. For our patient, ER and PR were both positif. Ninety hundred percent of oestrogen receptor and 70% of progesterone receptor. Immunochemical analysis of CAC has a debatable aspect.

Therefore, the hormone receptors are not discriminatory to distinguish CAC and metastasis lobular breast carcinoma, because apocrine glands expresses estrogen and progesterone receptors. It needs set of arguments. CK7 is not a discriminatory marking because it can be present in both CAC and in breast metastase. In this case, we opted for CAC due to clinical presentation in axilla of a hummoky tumor, the indolent clinical course and negativity explorations in search of another primitive neoplasy and decapitation secretion and attachment of neighborouing follicular epithelium caracteristis of apocrin glands in histology. Standard treatment is surgical excision with margins of 2 to 3cm for local tumor, for apocrine adenocarcinoma regional lymph node dissection if nodes were clinically positive is wide surgical excision. This kind of tumour is chemoresistant [16]. In this case, adjuvant chemotherapy was indicated, before surgery to reduce tumoral volume. Witold Kycler and al. [17] recommended chemotherapy (adriablastin and cyclophosphamide) and palliative treatment for metastatic apocrine adenocarcinoma of the axillary area.

We don’t dispose of much data for evolution, but a study of series treating apocrine adenocarcinoma since 1951, showed 39 were located in axilla, 12 patient had local relapse, 23 had lymph nodes relapse, 12 patients with visceral metastases and 9 patient dead [18]. We need more prospective studies to define precisely prognosis of such tumors that are still rare, so we can adopt a efficient diagnostic and treatment strategy.

## Conclusion

In conclusion, this case illustrates the importance clinicopathological correlation of skin cancer, particularly apocrine one. Clinical particularity and careful analyses histology helps diagnosis approach. More studies will help determining there is no treatment consensus. The worrying aspect in our histological patient should prompt careful clinical monitoring to detect a possible poor outcome.

## Competing interests

The authors declare no competing interests.
